# Structure and Chemical Organization in Damselfly *Calopteryx haemorrhoidalis* Wings: A Spatially Resolved FTIR and XRF Analysis with Synchrotron Radiation

**DOI:** 10.1038/s41598-018-26563-6

**Published:** 2018-05-30

**Authors:** Susan Stuhr, Vi Khanh Truong, Jitraporn Vongsvivut, Tobias Senkbeil, Yang Yang, Mohammad Al Kobaisi, Vladimir A. Baulin, Marco Werner, Sergey Rubanov, Mark J. Tobin, Peter Cloetens, Axel Rosenhahn, Robert N. Lamb, Pere Luque, Richard Marchant, Elena P. Ivanova

**Affiliations:** 1 Analytical Chemistry – Biointerfaces, Ruhr-University Bochum, Universitätsstraße 150, 44780 Bochum, Germany; 2 School of Science, Faculty of Science, Engineering and Technology, Swinburne University of Technology, PO Box 218, Hawthorn, Victoria 3122 Australia; 3 Infrared Microspectroscopy Beamline, Australian Synchrotron, 800 Blackburn Road, Clayton, Victoria 3168 Australia; 4 ID16A beamline, ESRF, The European Synchrotron, 71, avenue des Martyrs, 38000 Grenoble, France; 5 Departament d’Enginyeria Quimica, Universitat Rovira i Virgili, 26 Av. dels Paisos Catalans, 43007 Tarragona, Spain; 6 Advanced Microscopy Facility, Bio21 Institute, University of Melbourne, Parkville, Melbourn, Victoria 3010 Australia; 7 School of Chemistry, University of Melbourne, Parkville, Melbourne, Victoria 3010 Australia; 8 Museu de les Terres de l’Ebre, Gran Capità, 34, 43870 Amposta, Spain; 9 Melbourne Museum, GPO Box 666, Melbourne, 3001 Victoria Australia; 10 School of Science, RMIT University, Melbourne, Victoria 3001 Australia; 11 Canadian Light Source Inc., 44 Innovation Boulevard, Saskatoon, S7N 2V3 Canada

## Abstract

Insects represent the majority of known animal species and exploit a variety of fascinating nanotechnological concepts. We investigated the wings of the damselfly *Calopteryx haemorrhoidalis*, whose males have dark pigmented wings and females have slightly pigmented wings. We used scanning electron microscopy (SEM) and nanoscale synchrotron X-ray fluorescence (XRF) microscopy analysis for characterizing the nanostructure and the elemental distribution of the wings, respectively. The spatially resolved distribution of the organic constituents was examined by synchrotron Fourier transform infrared (s-FTIR) microspectroscopy and subsequently analyzed using hierarchical cluster analysis. The chemical distribution across the wing was rather uniform with no evidence of melanin in female wings, but with a high content of melanin in male wings. Our data revealed a fiber-like structure of the hairs and confirmed the presence of voids close to its base connecting the hairs to the damselfly wings. Within these voids, all detected elements were found to be locally depleted. Structure and elemental contents varied between wing membranes, hairs and veins. The elemental distribution across the membrane was rather uniform, with higher Ca, Cu and Zn levels in the male damselfly wing membranes.

## Introduction

Insects have evolved their ability to fly at least 400 million years ago and represent half of all living organisms on earth. Thus, understanding the structure and the composition of insect wings is fundamentally important^1,2^. Some species of the family of damselflies, *Calopterygidae*, in the order *Odonata*, are notable for their pigmented wings and striking body colors^3–5^. *Calopteryx haemorrhoidalis* with black wings inhabits Western Mediterranean, Algeria, France, Italy, Spain and Monaco^6–8^. The wings of mature male damselfly of this species are dark brown, while the female damselfly wings are lightly colored and appear to be translucent^9–11^. It was reported that melanin, which contains an essential amino acid phenylalanine, was the key compound responsible for the wing pigmentation^9–11^. One of the critical physiological functions of phenylalanine is the resistance to infection, which provides damselflies a unique ability to cope with parasite infection^9–12^. Previous studies also suggested that melanin present inside insect wings was in the form of eumelanin rather than in the form of pheomelanin^9,12^. In principle, the fundamental components of the cuticular layer of insect wings are (*i*) structural proteins and chitin that form the bulk of the wing membrane, and (*ii*) waxy components of the epicuticle composed of long chain aliphatic hydrocarbons (C_14_–C_30_) together with some carboxylic acids (palmitic acid and stearic acid)^13^. According to our previous synchrotron-Fourier transform infrared microspectroscopy (s-FTIR) investigation, the proportion of the major components of the insect wing membrane was found to be similar across different insect species^14,15^. However, to our knowledge, there is still a lack of crucial information on the elemental compositions of the insect wings.

The aim of this work was to gain a comprehensive understanding of chemical and elemental compositions in different parts of male and female wings of *Calopteryx haemorrhoidalis* using s-FTIR and synchrotron X-ray fluorescence (XRF) microscopy techniques. The highly collimated synchrotron light source essentially offered the combination of high brilliance, narrow bandwidth and nearly diffraction-limited focus, which exceptionally allows spatially resolved FTIR chemical mapping measurement and nanoscale elemental XRF investigation of the damselfly wings as reported in this study.

X-ray fluorescence microscopy is a multi-elemental analytical technique allowing chemical mapping not limited to the surface^16–19^. It allows the non-invasive analysis of the elemental composition of samples and the high penetration depth of about 800 µm at a photon energy of 17 keV provides bulk sensitive information. The spatial resolution of this technique was limited to approximately micrometer resolution due to the X-ray beam size. In the last decade, the X-ray focusing devices and the brightness of third generation synchrotron sources have advanced enough to produce intense sub-micrometer beams, which can be applied to map trace elemental distributions in biological samples at the sub-cellular scale^18,19^. The spatial resolution of XRF microscopy has been improved at the Advanced Photon Source (APS, Argonne, Illinois, USA) with the beam size reaching 150 nm × 150 nm ^16,17^. In the new Nano-Imaging beamline ID16A-NI at the European Synchrotron Radiation Facility (ESRF, Grenoble, France), an X-ray nanoprobe with a high flux of about 3 × 10^11^ photons s^−1^, can be focused down to the size of 25 (H) nm × 37 (V) nm^20,21^.

Pigment patterns of *Calopteryx haemorrhoidalis* are among the most striking and variable features^6,7^. Melanization is the pigmentation process wherein precursors (catecholamine) are converted into pigment molecules that are incorporated into the cuticle. There are a number of studies showing the incorporation of metals within the melanosomes in other organisms^22^. However, there is no information about the distribution of trace metal associated with melanin present in wing cuticle. The information would allow to extensively understand the role of melanin in insect and identify the significant role of melanin not only in kingdom of *Insect* but also other kingdoms, such as *Fungi* and *Animalia*. X-ray fluorescence microscopy based on the new X-ray nanoprobe allowed a unique pathway to characterize elemental distributions across the *Calopteryx haemorrhoidalis* wings, while functionality distributions were characterized by s-FTIR.

## Results

### Morphology of female and male *Calopteryx haemorrhoidalis* wings

**Figure 1:**
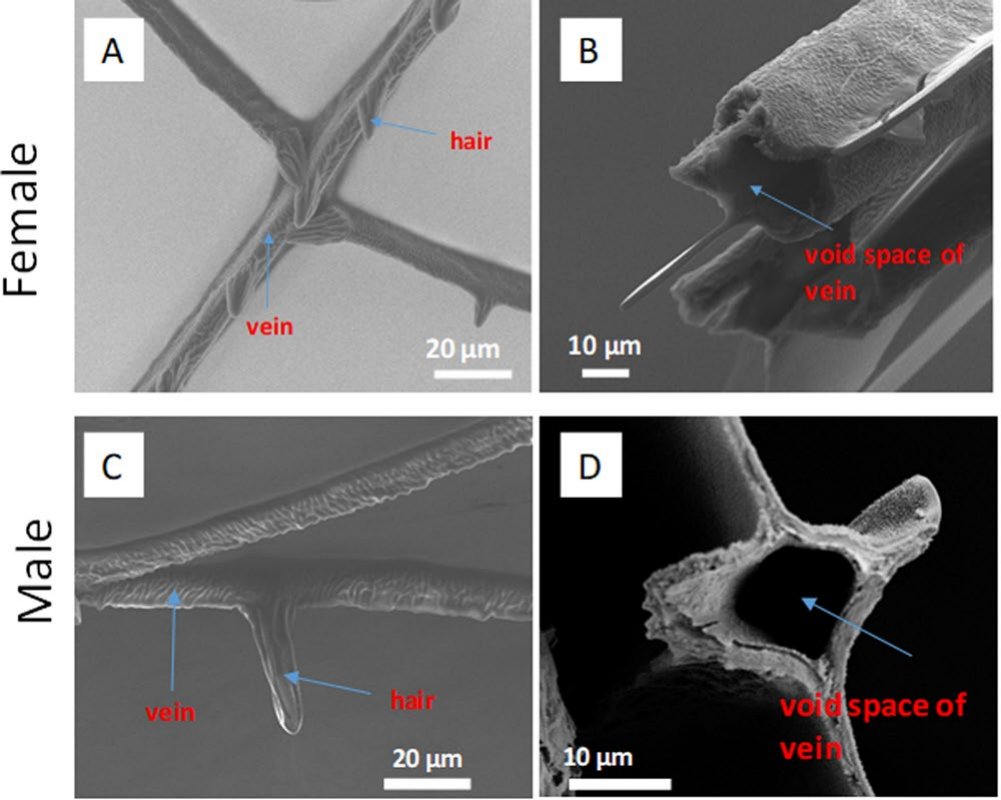
Morphology of female and male Calopteryx haemorrhoidalis wing membrane. Scanning electron micrographs (left) showing veins and hairs on the membrane of (**A**) female and (**C**) male wings. Cross-sections (right) showing the void space in the veins on the membrane of (**B**) female and (**D**) male wings.

SEM was applied to visualize the wing membrane structure of the female and male *Calopteryx haemorrhoidalis*, as shown in Fig. 1 and Supplementary Fig. S1. In particular, the low-vacuum SEM technique used in Supplementary Fig. S2 allowed the imaging of damselfly wings to be performed without the use of gold coating on the samples. This technique was used in order to rule out the influences of high vacuum and gold-coating. The data presented in Supplementary Fig. S2 confirmed the unique morphology of the female and male *Calopteryx haemorrhoidalis* wings. On the male and female damselfly wing membranes, there are three typical regions of interest including the supporting structures in the form of the thicker filaments with diameters in the order of 5 µm, thin hairs and the membrane that covers the wing (Fig. 1 and Supplementary Fig. S1). This morphology is characteristic of insect wings^23,24^. Typical cross sections of the wing veins are shown in Fig. 1B,D for female and male damselfly wings, respectively. The length of the hairs observed on the wing membranes were found to be in the range of 27.1 ± 3.4 µm and 28.0 ± 4.3 µm for the female and male wings, respectively. Figure 1A,C reveal that the base of the hair that originated from the veins contained a cavity, which is formed during growth when a damselfly changes from a larva to an adult. Damselfly wings are usually quite soft and rumpled when the larvae emerge from the water. They then expand their wings by pumping a body fluid known as hemolymph through the veins in the wing. Once the wings are fully expanded, the hemolymph is withdrawn and the veins harden^25^. The result from SEM analysis also indicated that there was no significant difference between fore- and hind-wings of *Calopteryx haemorrhoidalis* observed from the same gender (Supplementary Fig. S3).

### Microscale chemical characterization of the female and male *Calopteryx haemorrhoidalis* wing membrane

**Figure 2:**
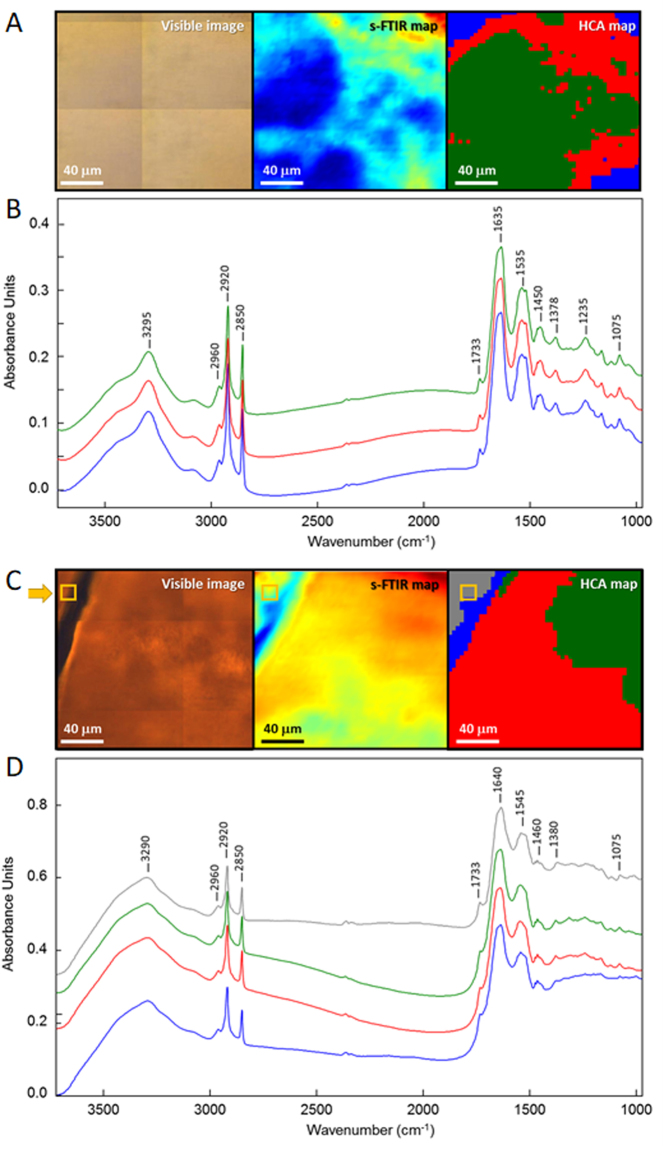
s-FTIR analysis of female and male Calopteryx haemorrhoidalis wing membranes. Comparison of visible image, s-FTIR chemical map of protein distribution (amide I), and hierarchical cluster analysis (HCA) of (**A**) female and (**C**) mature male wings. Average representative s-FTIR spectra of each color-coded area in the HCA map for (**B**) female and (**D**) mature male wings.

To gain an insight into the chemical nature of female and male damselfly wings (both mature), high-resolution s-FTIR maps were acquired at the center of the wing membrane at the location similar to that shown in Fig. 2. Our initial examination of the spectral features was made by comparing average s-FTIR spectra extracted from fore- and hind-wing membranes of male and female *Calopteryx haemorrhoidalis*, which were shown to be closely similar within the same gender (Supplementary Fig. S4). Such a strong similarity suggested that there was no significant difference in the chemical compositions between fore- and hind-wing membranes observed within the same gender. In addition, the average spectra obtained from the male damselfly wing membrane clearly indicated the characteristic features of melanin, which are typically presented as strong broad bands in the low-wavenumber region (Supplementary Fig. S5), suggesting a substantial amount of melanin in the male damselfly wing. The spectra of the female damselfly wing, on the contrary, showed the absence of the melanin signatures, which suggested a lack or only a minimal amount of melanin existed. Fig. 2A,C presented the visible image of the wing membrane and the corresponding protein distribution based on the integrated area under amide I band at 1635 cm^−1^ in the s-FTIR spectral maps observed for female and male damselfly wings, respectively. The pattern recognition approach, specifically hierarchical cluster analysis (HCA), was subsequently performed on the s-FTIR maps to examine the uniformity (similarity) of the chemical distribution in the female and male wings. The HCA discriminated the spectra into three groups as illustrated by the three color-coded regions. The average spectra, which were calculated from each group (Fig. 2B,D), appeared to be very similar with only a subtle difference in the band ratios of carbonyl lipid and amide I protein at 1733 cm^−1^ and 1635 cm^−1^, respectively. Overall, this indicated a uniform chemical distribution across the wing membrane. It should also be noted that there is no significant evidence of melanin in the s-FTIR spectra of the female wing membrane when compared to the synthetic melanin spectra in which the characteristic broad overlapping band features are present in the range of 1400–1000 cm^−1^ (Supplementary Fig. S5).

**Figure 3:**
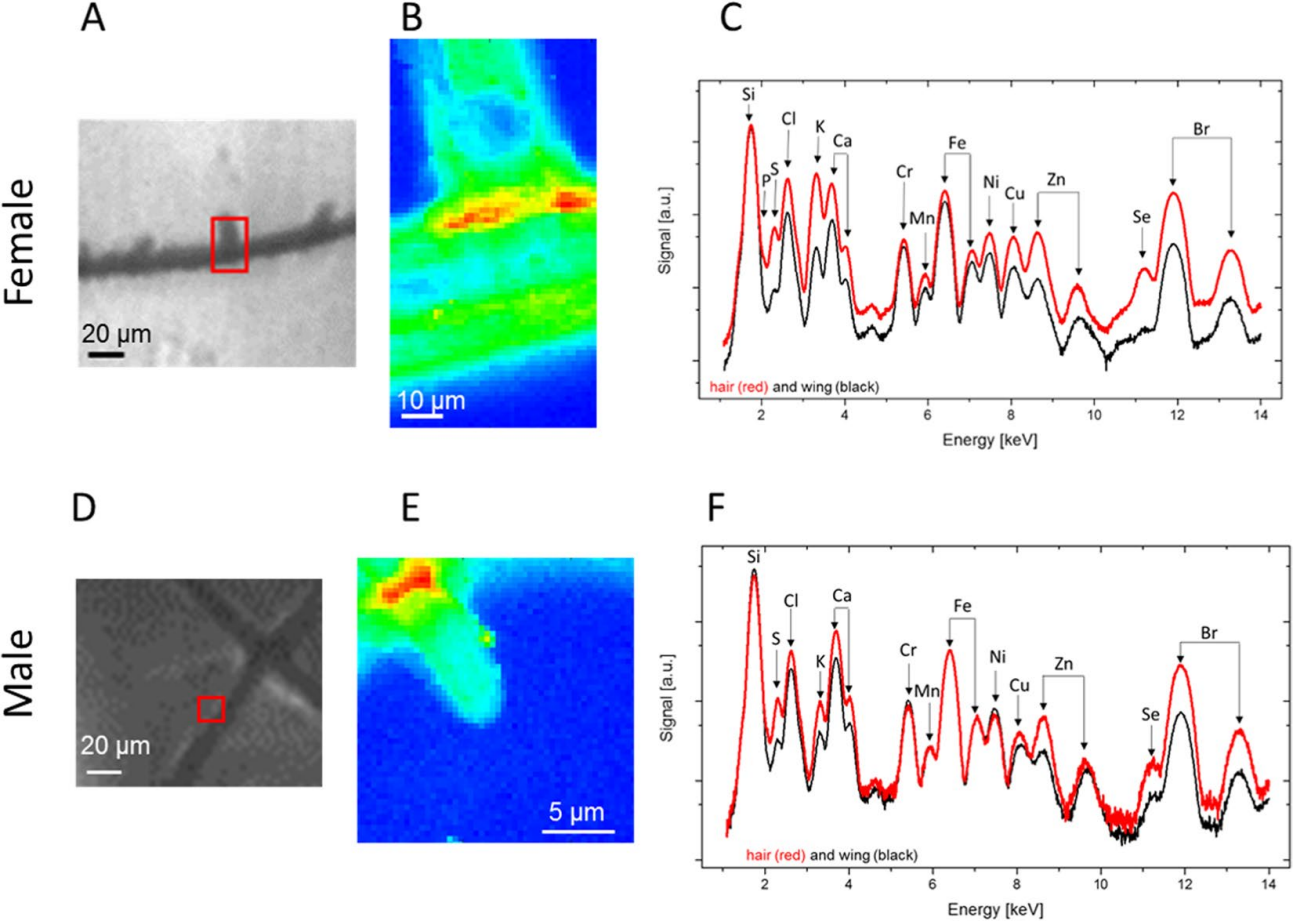
Chemical characteristics of the female and mature male Calopteryx haemorrhoidalis wing membranes as determined by nanoprobe XRF. Microscopy image of the wing of the (**A**) female and (**D**) male damselfly with the scanned area marked as red square. Total fluorescence yield of the scanned area for the (**B**) female and (**E**) male damselfly wings. The blue area represent the wing membrane, while the red and green parts were obtained on the hair structure. Accumulated XRF spectra of the region of the hair (red) and of the wing (black) for (**C**) female and (**F**) male damselfly wings.

According to the resultant HCA map, the top grey spectrum in Fig. 2D is the average representative s-FTIR spectrum of the wing in the area near the base of the hair spike, similar to the area used in the XRF measurement in Fig. 3. Unlike the female damselfly wing, the difference in spectral features between the four color-coded areas on the male wing are essentially the broad overlapping bands in 1400–1000 cm^−1^ spectral range, which are characteristic for melanin pigments. Such spectral evidence not only points out substantial amounts of melanin present across the male wing, but also suggests that there may be different combinations of melanin molecules on different areas of the wing (*i*.*e*. vein, membrane and the interface between the vein and the membrane). In addition to the strong characteristic melanin features, a slight shift of the amide I band towards higher wavenumber (Δ$\bar{{\nu }}$ = +5 cm^−1^) was observed in the male damselfly wing compared to those of the female wing. This chemical shift of amide I could be due to hydrogen-bonding interactions between carbonyl groups of melanin and amide groups of protein molecules in the wing membrane.

### Nanoscale elemental mapping of the female and male *Calopteryx haemorrhoidalis* wing membrane

**Figure 4:**
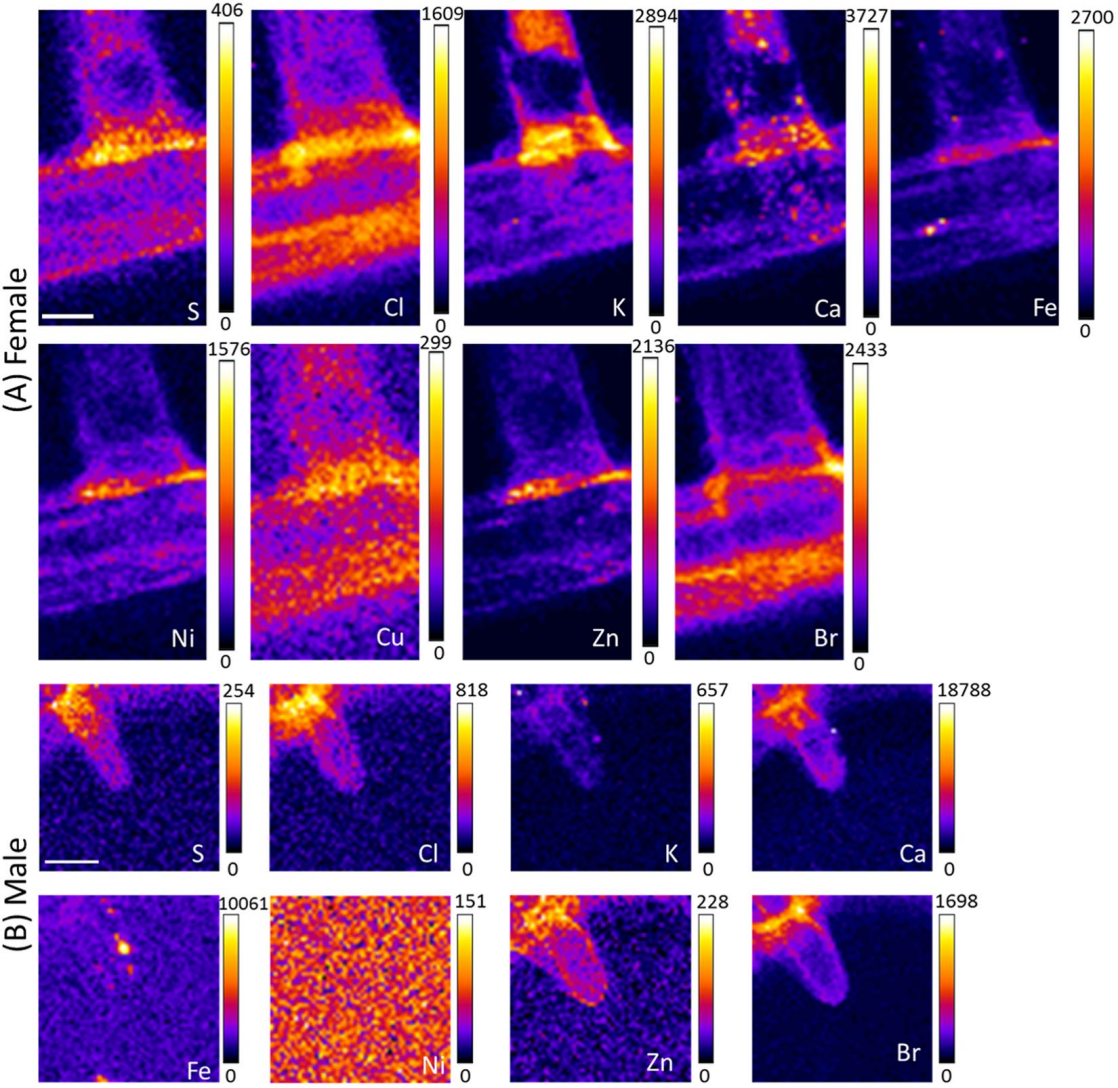
Elemental distribution across the female and male Calopteryx haemorrhoidalis wings. XRF analysis of the (**A**) female and (**B**) mature male damselfly wings (scale bar 5 µm). While 4A corresponds to 3A, 4B belongs to 3B. The concentration of the elements is given in µg/µm^2^.

In Fig. 3A,D, the membrane areas on both female and male *Calopteryx haemorrhoidalis* wings were marked to indicate where spatially resolved XRF data was obtained. The normalized total fluorescence yield maps in Fig. 3B,E show higher material densities in the area of the vein and the hair structure. A comparison of the fluorescence yield in the area of the hair indicated a higher material density as compared to the membrane. For both female and male species, elements including P, S, Cl, K, Ca, Cr, Mn, Fe, Ni, Cu, Zn, Se, and Br were detected (Figs. 3C,F). It should be noted that the strong Si signal originated from the silicon nitride membranes, which were used as a supporting material for the wings during the measurement. For both female and male damselfly wings, the concentrations of Br and S were very similar in the hair and the wing membrane. The veins of female and male wings showed depleted material densities inside the core structure, confirming the hollow fiber-like cavity presented in the SEM cross-section images (Fig. 1). It is likely that these hollow structures were formed in the larval stage before the veins were hardened. The spatially resolved XRF images in Fig. 4A,B show higher concentrations of S, Cl, Ca and Br in the areas close to the junction of the supporting structure. A quantification of the density of the elements for male and female species and the comparison of hair and membranes is provided in Table 1. Most of the elements in the female hair were higher or similar in concentration to those in the male hair, except for Ca (468.3 µg/µm^2^ *vs*. 749.6 µg/µm^2^). Elevated metal levels were detected in the female hair for K (484.0 µg/µm^2^ *vs*. 79.0 µg/µm^2^), Ni (220.0 µg/µm^2^ *vs*. 91.3 µg/µm^2^), and Zn (208.8 µg/µm^2^ *vs*. 86.6 µg/µm^2^). The membrane material of the male damselfly contains higher or similar metal levels compared with those in the female one. Enhanced levels were detected in the male membrane for S (25.2 µg/µm^2^ *vs*. 11.1 µg/µm^2^), Cl (156.8 µg/µm^2^ *vs*. 93.4 µg/µm^2^), Ca (252.8 µg/µm^2^ *vs*. 60.0 µg/µm^2^), Ni (88.0 µg/µm^2^ *vs*. 43.4 µg/µm^2^), Cu (34.4 µg/µm^2^ *vs*. 23.6 µg/µm^2^), Zn (33.8 µg/µm^2^ *vs*. 23.3 µg/µm^2^), and Br (95.5 µg/µm^2^ *vs*. 86.4 µg/µm^2^) (Table 1). Our nanoscale XRF results indicate a distinction of elemental compositions of the different structures present in the wings of male and female damselflies.

**Table 1:** Comparison of average concentration of elements between female and male Calopteryx haemorrhoidalis wings, observed on the areas of hair (top) and membrane (bottom).

	Element concentration [µg/µm^2^]	
	Female hair	Male hair
Sulfur	81.4 ± 8.4	71.8 ± 5.7
Chlorine	459.7 ± 43.0	329.9 ± 20.1
Potassium	484.0 ± 62.0	79.0 ± 5.0
Calcium	468.3 ± 59.2	749.6 ± 54.7
Nickel	220.1 ± 23.1	91.3 ± 2.2
Copper	86.9 ± 7.9	57.1 ± 2.6
Zinc	208.8 ± 27.4	86.6 ± 5.0
Bromium	499.6 ± 36.6	450.1 ± 38.8
**Female membrane Male membrane**		
Sulfur	11.1 ± 1.2	25.2 ± 1.2
Chlorine	92.4 ± 9.2	156.8 ± 3.5
Potassium	35.3 ± 3.6	33.8 ± 1.6
Calcium	60.0 ± 7.0	252.8 ± 6.5
Nickel	43.4 ± 5.0	88.0 ± 2.1
Copper	23.6 ± 2.8	34.4 ± 1.6
Zinc	23.3 ± 2.4	33.8 ± 1.5
Bromium	86.4 ± 7.4	95.5 ± 3.2

## Discussion

It is commonly known that the damselfly wings possess a number of sophisticated characteristics involving their super-hydrophobic, self-cleaning and bactericidal properties^26–28^, even though the chemical compositions of the nanostructures responsible for such behaviors are yet to be fully understood. In particular, the differences between pigmented and non-pigmented species have to be investigated. The fact that Zn, Cu, Fe and Ca were in many cases simultaneously present at distinct parts of the wings is in line with the recent observation that there are many biological examples in which Zn, Cu, and Fe are correlated and Mn is frequently found in conjunction with Ca^29^. Several metals like Zn, Mn, Fe and Ca were previously found to be concentrated within the cuticle of mouth hooks, claws, mandibles and ovipositors. It was found out that Zn and Cl correlate with the hardness and elastic modulus as higher levels of Zn enhanced hardness by 20%^30^. Such a mechanical influence on the elemental composition might also be present in damselfly wings, which could imply different elastic properties of the male and female wings.

**Figure 5:**
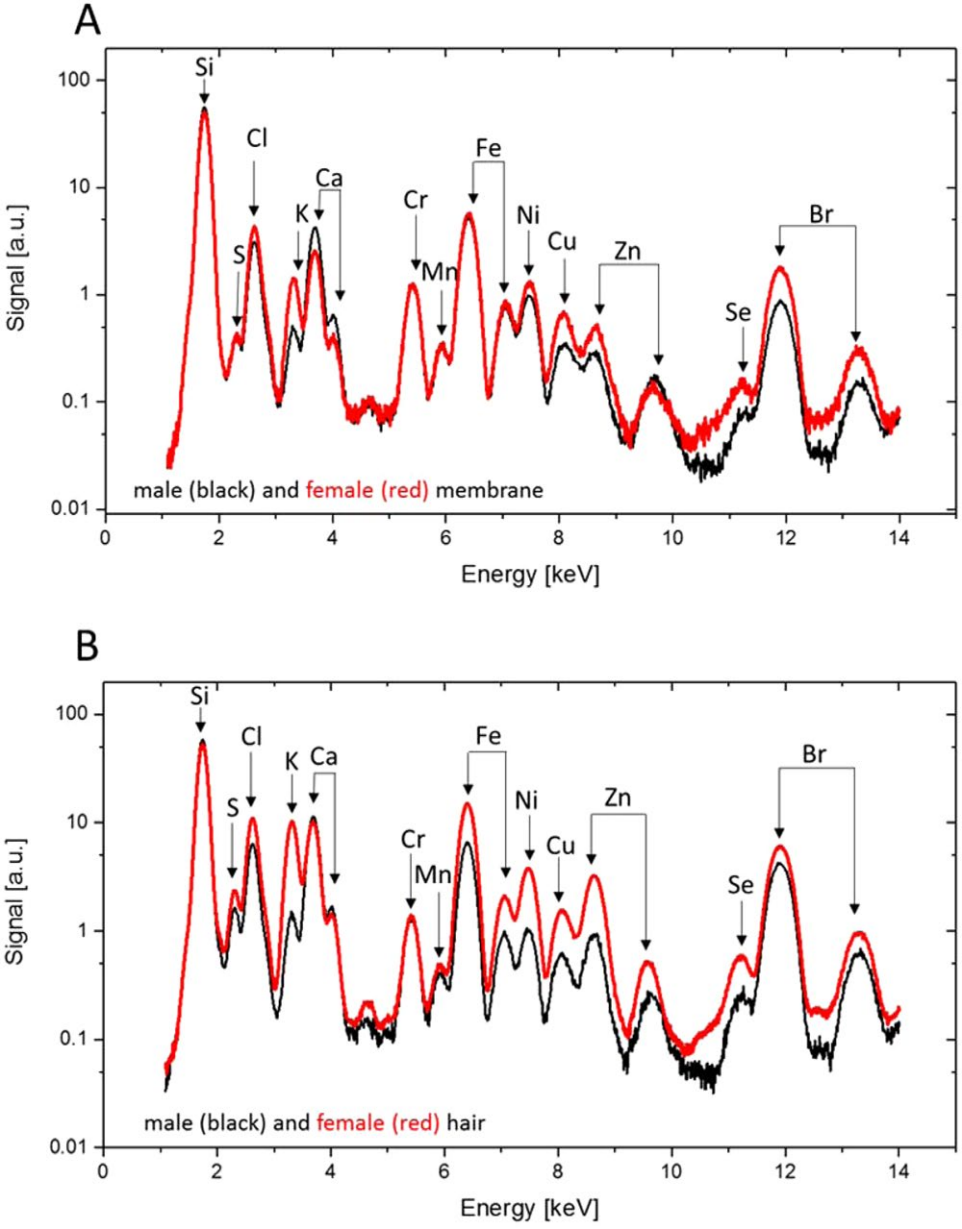
Comparison of elements between female and male damselfly wings on the membrane and hair areas. XRF spectra of the (**A**) membrane and (**B**) hair of a female (red) and male (black) Calopteryx haemorrhoidalis wings.

Comparison of the wing membranes of the pigmented male and the non-pigmented female indicated several differences (see Fig. 5A and Table 1). While the amounts of K were insignificantly different between male and female wings (33.8 *vs*. 35.3 µg/µm^2^ in male and female wings, respectively), the amounts of Cl (156.8 µg/µm^2^ *vs*. 93.4 µg/µm^2^) and Ca (252.8 µg/µm^2^ *vs*. 60.0 µg/µm^2^) were substantially higher in the male wing. In addition, the male wing membrane contained more copper and zinc (34.4 µg/µm^2^ and 33.8 µg/µm^2^ in the male wing, compared to 23.6 µg/µm^2^ and 23.3 µg/µm^2^ in the female wing). Since melanin contains substantial amounts of Cu and Zn^31,32^, the higher metal readings could be indicative of higher melanin concentrations in the male damselfly wing. Thus, our results support the key role of melanin in the visual, stronger pigmentation of the male damselfly wings (e.g. with high eumelanin contents)^22,31,32^.

## Conclusion

In this study, the chemical compositions of the damselfly *Calopteryx haemorrhoidalis* wings were investigated. While a low concentration of melanin and a uniform chemical distribution were observed for the female wings using s-FTIR, the male wings showed substantially higher melanin contents. Differences in the elemental compositions were observed between the two genders. The nanoscale XRF data revealed that the concentrations of Cu, Zn and Ca were substantially higher in the male wing membranes. The results obtained from SEM and XRF analysis clearly indicate the distinct morphology and its connection with chemistry and elemental compositions of hairs and membranes of the male and female damselfly wings. The combination of the different state-of-art techniques that provide complementary, spatially resolved data is a powerful tool to understand the chemical organization in biological systems. In particular, new technical implementations currently underway will allow the full three-dimensional analysis of biological systems in the near future.

## Material and Methods

### Preparation of damselfly wing samples

*Calopteryx haemorrhoidalis*^33^ damselfly specimens were collected from Els Ports Natural Park in Catalonia, Spain. The membranes of the damselfly wings on the dorsal side of their forewing were dissected into the square sections of approximately 5 mm × 5 mm using a surgical blade. The wing sections were then gently rinsed with MilliQ H_2_O (resistivity of 18.2 MΩ cm^−1^, Merck Millipore, Washington, DC, USA) and then blow-dried using 99.99% purity nitrogen gas, as described elsewhere^26,34^.

### Scanning electron microscopy

Scanning electron microscopic (SEM) images were collected using a field-emission FEI Nova NanoSEM (ThermoFischer Scientific, Hillsboro, USA) at 3 kV. Prior to the measurement, the wing samples were sputter coated with a thin layer of gold (approximately 3 nm in thickness) using a Dynavac CS300 coating unit (Australia). Quantitative analysis of the SEM images was carried out using ImageJ software (NIH USA Image ver. 1.51), over 30 different spots from 2 images. There are two insect specimens of male and female examined for SEM analysis. FEI Teneo VolumeScope environmental SEM (ThermoFischer Scientific, Hillsboro, USA) was operated at 5 kV and 0.1 nA under the low vacuum to image the whole wings without the assistance of gold-coatings.

### Synchrotron Fourier transform infrared (s-FTIR) microspectroscopy

The s-FTIR measurement was performed at the Infrared Microspectroscopy (IRM) beamline of the Australian Synchrotron using a Bruker Vertex V80 v spectrometer coupled with a Hyperion 2000 FTIR microscope (Bruker Optik GmbH, Ettlingen, Germany) and equipped with a liquid nitrogen-cooled narrow-band mercury cadmium telluride detector. The spatially resolved distribution of the chemical functional groups present on the damselfly wings was mapped and characterized in transmission mode at a high resolution, which was critically required for surface characterization of the wing samples. In this study, the synchrotron transmission measurement was performed using a 36× IR objective (NA = 0.50; Bruker Optik GmbH, Ettlingen, Germany) with the aperture size adjusted to 4 × 4 µm^2^ for every pixel, and the spectra were acquired at a 4-µm step interval between pixels.

In practice, the wing section was held on a gap between aluminum support frames using polyimide (Kapton^®^) tape to fix both sides of the wing section. The s-FTIR chemical maps were then acquired to cover an area of 160 × 160 μm^2^ on the wing. For each pixel, the s-FTIR spectrum was recorded within a spectral range of 3800–700 cm^−1^ using 4 cm^−1^ spectral resolution and 16 co-added scans. Blackman-Harris 3-Term apodization, Power-Spectrum phase correction, and zero-filling factor of 2 were set as default acquisition parameters using OPUS 7.2 software suite (Bruker). Two IR maps have been on acquired on fore- and hind-wings of one insect specimen. There are two insect specimens of male and female examined for IR mapping.

### Spectral pre-processing and hierarchical cluster analysis (HCA)

Hierarchical cluster analysis (HCA) was performed on the acquired s-FTIR maps using CytoSpec v. 1.4.02 (CytoSpec Inc., Boston, MA, USA), as previously described^35,36^. The s-FTIR absorbance spectra embedded in each chemical map were first pre-processed with a noise reduction algorithm before being transformed into 2^nd^ derivative spectra using Savitzky-Golay algorithm with 13 smoothing points. After that, the 2^nd^ derivative spectra were normalized using extended multiplicative signal correction (EMSC)^37^. HCA was subsequently performed on the pre-processed spectral map using two spectral regions of 3020–2800 and 1830–1000 cm^−1^.

### Synchrotron X-ray fluorescence (XRF) measurement

For the synchrotron nano-XRF measurements one specimens was investigated from a male and one specimen from a female damselfly. To prepare the wing samples for the XRF measurements, the wings of the damselflies were broken with a tweezer and subsequently sandwiched between two silicon nitride membranes (500 nm membrane thickness, the window size is 1.5 × 1.5 mm^2^, Silson Ltd, Warwickshire, England). The samples were measured under vacuum at the Nano-Imaging beamline ID16A-NI at the European Synchrotron Radiation Facility (ESRF, Grenoble, France) using an excitation photon energy of 17 keV. The fluorescence detector (six element silicon drift detector, SGX SENSORTECH) was placed next to the sample, perpendicular to the beam. The beam focus was 42 × 39 nm^2^, while the photon flux during the measurements was 7·10^10^ ph/s. A step size of 400 nm and a dwell time of 0.1 s were used for scanning the regions of interest. The analysis of the data was performed with the X-ray fluorescence toolkit PyMCA^38^. The quantitative results were calibrated using a Thin Film XRF Reference Sample (RF7-200-S2371, Axo Dresden GmbH, Dresden, Germany).

## Electronic supplementary material

Supplementary Information
