# Supplementary material for: Structure and Chemical Organization in Damselfly Calopteryx haemorrhoidalis Wings: A Spatially Resolved FTIR and XRF Analysis with Synchrotron Radiation
Source: Sci Rep. 2018 May 30;8:8413. doi: 10.1038/s41598-018-26563-6 (PMC5976759; doi:10.1038/s41598-018-26563-6)
Supplement: Supplementary file 1 — Supplementary Information [file 41598_2018_26563_MOESM1_ESM.pdf]

SUPPLEMENTARY INFORMATION

Structure and Chemical Organization in Damselfly *Calopteryx haemorrhoidalis* Wings: A Spatially Resolved FTIR and XRF Analysis with Synchrotron Radiation

Susan Stuhr[1], Vi Khanh Truong[2], Jitraporn Vongsvivut[3], Tobias Senkbeil[1], Yang Yang[4], Mohammad Al Kobaisi[2], Vladimir A. Baulin[5], Marco Werner[5], Sergey Rubanov[6], Mark J. Tobin[3], Peter Cloetens[4], Axel Rosenhahn[1], Robert N. Lamb[7], Pere Luque[8], Richard Marchant[9] & Elena P. Ivanova[2,10*]

[1]Analytical Chemistry – Biointerfaces, Ruhr-University Bochum, Universitätsstraße 150, 44780 Bochum, Germany

[2]School of Science, Faculty of Science, Engineering and Technology, Swinburne University of Technology, PO Box 218, Hawthorn, Victoria 3122, Australia

[3]Infrared Microspectroscopy Beamline, Australian Synchrotron, 800 Blackburn Road, Clayton, Victoria 3168, Australia

[4]European Synchrotron Radiation Facility, 71, avenue des Martyrs, CS 40220, 38043 Grenoble Cedex, France

[5]Departament d'Enginyeria Quimica, Universitat Rovira i Virgili, 26 Av. dels Paisos Catalans, 43007 Tarragona, Spain

[6]Advanced Microscopy Facility, Bio21 Institute, University of Melbourne, Parkville, Victoria 3010, Australia

[7]School of Chemistry, University of Melbourne, Parkville, Victoria 3010, Australia

[8]Museu de les Terres de l'Ebre, Gran Capità, 34, 43870 Amposta, Spain

[9]Melbourne Museum, 11 Nicholson St, Carlton, Victoria 3053, Australia

[10] School of Science, RMIT University, Melbourne, Victoria 3001, Australia

E-mail: elena.ivanova@rmit.edu.au

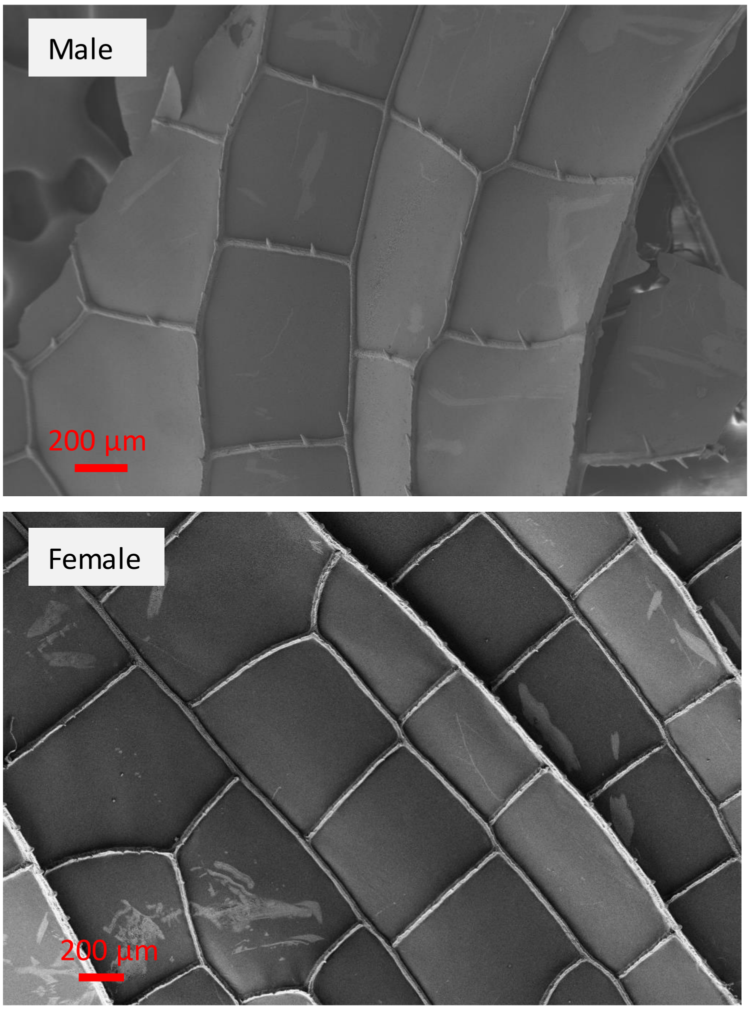

Figure S1. Overview of the male and female *Calopteryx haemorrhoidalis* wing membranes. Scanning electron micrographs showing the veins and hairs on the membrane of male and female wings.

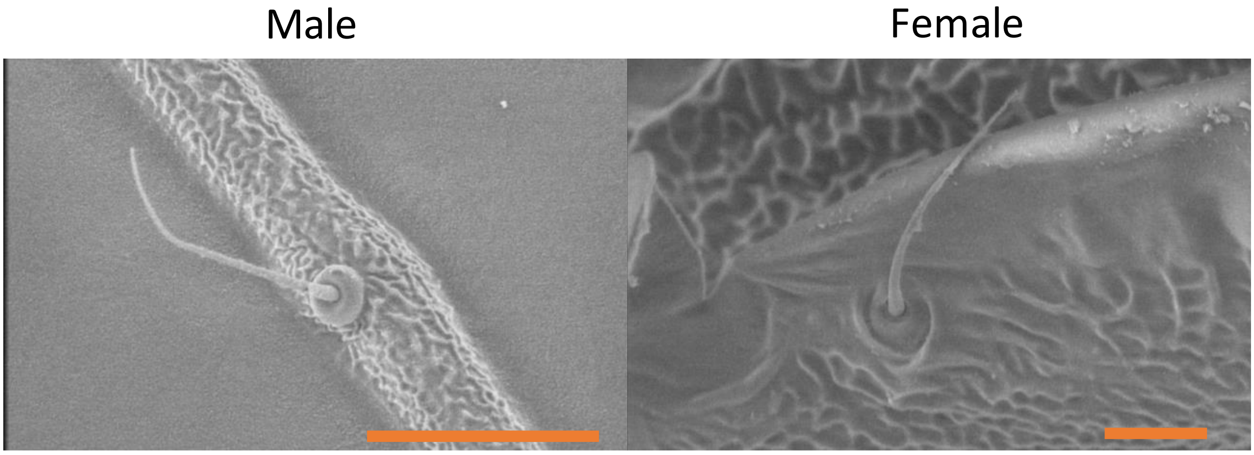

Figure S2. Vein and hair of male and female *Calopteryx haemorrhoidalis* wing membranes of fore- and hind-wings imaged under low vacuum SEM (Scale bar 20 µm).

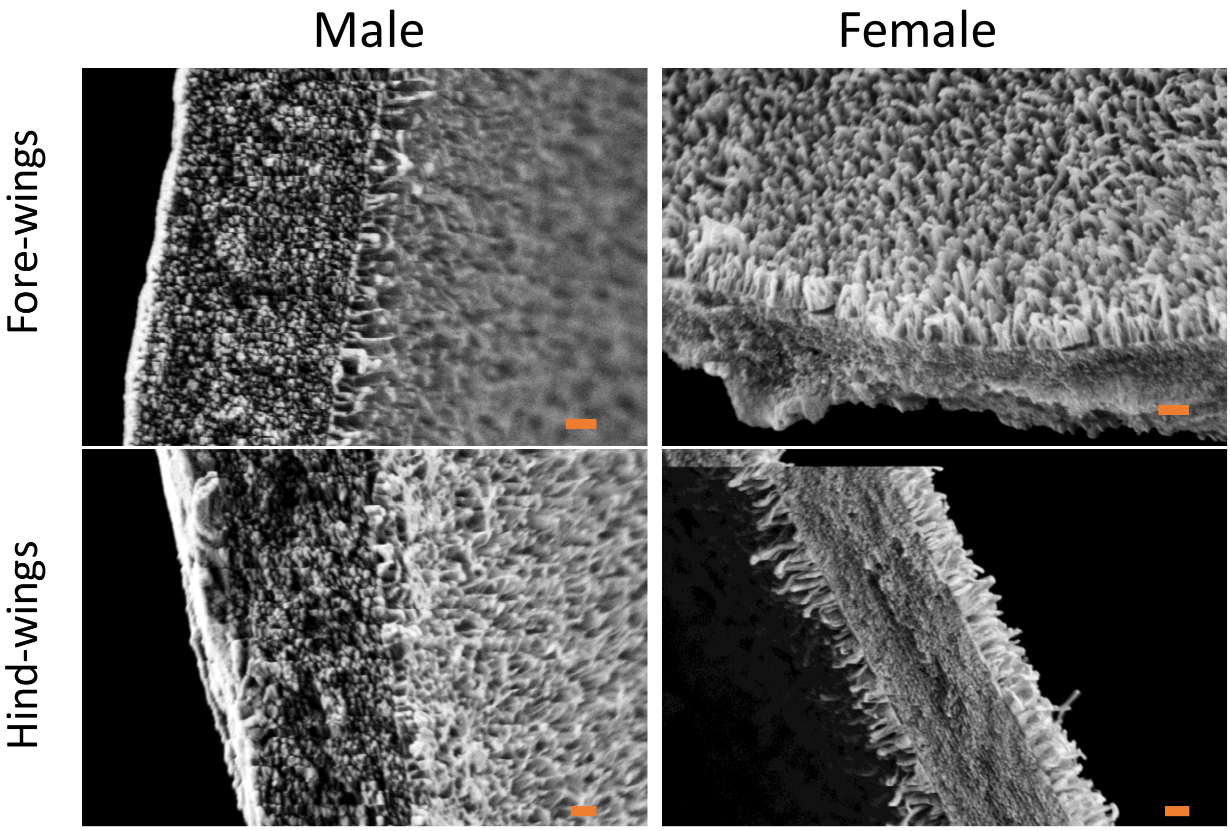

Figure S3. Scanning electron micrographs of the fore- and hind-wing membranes of male and female *Calopteryx haemorrhoidalis* (Scale bar 200 nm).

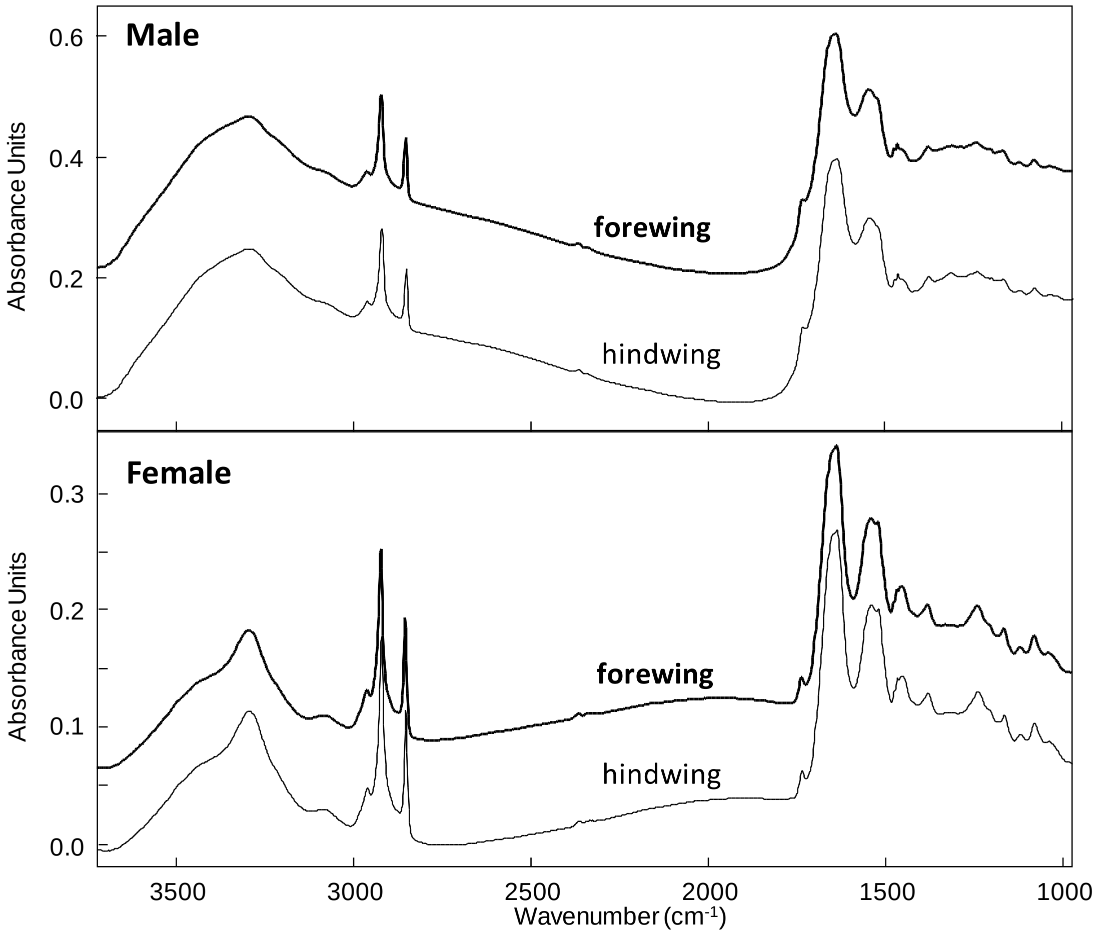

Figure S4. Average s-FTIR spectra obtained from the fore- and hind-wing membranes of male and female *Calopteryx haemorrhoidalis*.

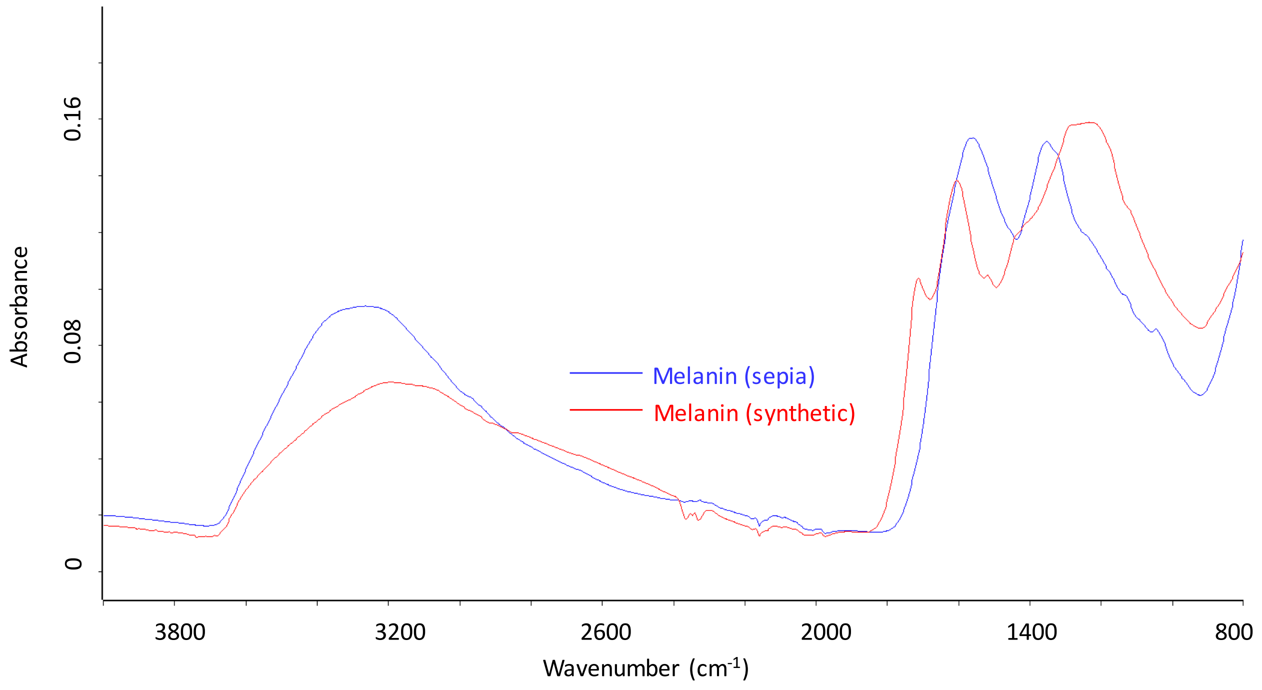

Figure S5. Reference ATR-FTIR spectra of the natural melanin exacted from *Sepia officinalis*, in comparison to the synthetic melanin produced by the oxidation of tyrosine with hydrogen peroxide.
